# Multidrug-resistant tuberculosis clusters and transmission in Taiwan: a population-based cohort study

**DOI:** 10.3389/fmicb.2024.1439532

**Published:** 2024-09-18

**Authors:** Kuang-Hung Liu, Yu-Xin Xiao, Ruwen Jou

**Affiliations:** ^1^Tuberculosis Research Center, Centers for Disease Control, Ministry of Health and Welfare, Taipei, Taiwan; ^2^Reference Laboratory of Mycobacteriology, Centers for Disease Control, Ministry of Health and Welfare, Taipei, Taiwan

**Keywords:** *Mycobacterium tuberculosis*, tuberculosis, whole genome sequencing, multidrug-resistance, transmission, mutation

## Abstract

**Introduction:**

Multidrug-resistant tuberculosis (MDR-TB) remains a challenge in the TB program of Taiwan, where 0.5% of new cases and 2.1% of previously treated cases were resistant to at least rifampin (RIF) and isoniazid (INH). Since >80% of our MDR-TB are new cases, genotyping of MDR *Mycobacterium tuberculosis* is implemented to facilitate contact investigation, cluster identification, and outbreak delineation.

**Methods:**

This is a population-based retrospective cohort study analyzing MDR-TB cases from 2019 to 2022. Whole genome sequencing (WGS) was performed using the Illumina MiSeq and analyzed using the TB Profiler. A single nucleotide polymorphism (SNP) threshold of ≤ 12 and phylogenetic methods were used to identify putative transmission clusters. An outbreak was confirmed using genomic data and epidemiologic links.

**Results:**

Of the 297 MDR-TB cases, 246 (82.8%), 45 (15.2%), and 6 (2.0%) were simple MDR, extensively drug-resistant tuberculosis (pre-XDR-TB) and extensively drug-resistant tuberculosis (XDR-TB), respectively. The sublineage 2.2 modern Beijing was the predominant (48.8%) MDR-TB strain in Taiwan. Phylogenetic analysis identified 25.3% isolates in 20 clusters, with cluster sizes ranging from 2 to 13 isolates. Nevertheless, only 2 clusters, one household and one community, were confirmed as outbreaks. In this study, we found that males had a higher risk of MDR-TB transmission compared to females, and those infected with the sublineage 2.1-proto-Beijing genotype isolates were at a higher risk of transmission. Furthermore, 161 (54.2%) isolates harbored compensatory mutations in the *rpoC* and non-rifampicin resistant determinant region (non-RRDR) of the rpoB gene. MDR-TB strains containing rpoB S450L and other compensatory mutations concurrently were significantly associated with clusters, especially the proto-Beijing genotype strains with the compensatory mutation *rpoC* E750D or the modern Beijing genotype strains with *rpoC* D485Y/*rpoC* E1140D.

**Discussion:**

Routine and continuous surveillance using WGS-based analysis is recommended to warn of risks and delineate transmission clusters of MDR-TB. We proposed the use of compensatory mutations as epidemiological markers of M. tuberculosis to interrupt putative MDR-TB transmission.

## 1 Introduction

The global emergence of multidrug-resistant tuberculosis (MDR-TB) poses a threat to achieving the End TB targets. MDR-TB can arise from delayed diagnosis, prolonged treatment, and the transmission of MDR *Mycobacterium tuberculosis* isolates. Although defining and tracking TB transmission has been particularly challenging due to the variability in disease progression after infection, genotyping of *M. tuberculosis* provides vital insight into investigating TB transmission patterns and aids in identifying associated risk factors (Gardy et al., 2011; Lalor et al., 2018).

Understanding these transmission patterns is crucial for guiding effective public health interventions aimed at preventing the spread of MDR-TB (Nathanson et al., 2010). Furthermore, identifying the risk factors associated with MDR-TB clustering is crucial for improving health outcomes through coordinated efforts to prevent transmission (Denholm et al., 2024; GBD 2021 Forecasting Collaborators, 2024). Compared to conventional genotyping methods (Barnes and Cave, 2003; Allix-Béguec et al., 2008), whole genome sequencing (WGS) provides a more accurate resolution of phylogenetic clusters, making it a powerful tool for TB surveillance and control (Luo et al., 2014; Yang et al., 2017; Guthrie et al., 2018, 2019).

Rifampicin-resistant (RR) isolates exhibit mutations within the RNA polymerase (*rpoB*) gene, with *rpoB* S450L being the most prevalent mutation (Comas et al., 2011; Brandis and Hughes, 2013). These RR isolates harbor compensatory mutations in RNA polymerase subunits, such as the *rpoA* and *rpoC* genes, or in regions outside the *rpoB* rifampin-resistance determining region (RRDR; non-RRDR). Specific mutations in the *rpoA* and *rpoC* genes can result in high-fitness MDR strains (Comas et al., 2011).

However, the relationship between drug resistance and compensatory mutations has been inconsistently reported (Cohen and Murray, 2004). Previous studies revealed that compensatory evolution can facilitate the spread of MDR *M. tuberculosis* isolates by mitigating the fitness costs associated with mutations, thereby increasing resistance rates (de Vos et al., 2013; Li et al., 2016; Merker et al., 2018). Conversely, other studies have indicated that compensatory mutations have a minimal impact on MDR-TB clustering in China (Liu et al., 2018; Chen et al., 2022).

The prevalence of MDR-TB in Taiwan is estimated to be over 80% (Chuang et al., 2016; Wu et al., 2023), yet the pattern of transmission remains unclear. To enhance the management of MDR-TB, we conducted a cohort study using WGS and epidemiological information to investigate putative MDR-TB transmission.

## 2 Materials and methods

### 2.1 Study design and population

In our TB program, bacteriological examination is required for any presumptive TB cases, with universal drug susceptibility testing (DST) achieved through the TB laboratory network. This population-based retrospective study analyzed culture-confirmed MDR-TB cases reported to the Taiwan Centers for Disease Control (Taiwan CDC) between 2019 and 2022. For each case, one initial *M. tuberculosis* isolate was analyzed using WGS.

*M. tuberculosis* isolates were cultured and manipulated in a certified biosafety level 3 laboratory. Demographic information, bacteriological data, and epidemiological investigation information were obtained from the National TB Registry. A new case was defined as one that had never been previously reported or recorded in the TB Registry as an MDR-TB case. Previously treated MDR-TB cases included those with recurrent cases, those treated after loss to follow-up, those treated after failure, and other previously treated cases. We used a standardized questionnaire to investigate epidemiological links among MDR-TB cases.

### 2.2 Mycobacterial culture, identification, and drug susceptibility testing

Decontaminated specimens were inoculated on both solid and liquid media simultaneously. *M. tuberculosis* isolates were subjected to DST using the agar proportion method (APM) with 7H10 and 7H11 media (Coning Technology Limited Company, Taiwan). The APM procedure is described as follows: *M. tuberculosis* was grown in 7H9 complete media [0.2% glycerol, 0.1% Tween, 10% albumin, dextrose catalase (ADC) supplement] to log phase. Cultures were adjusted to 0.5–1 MacFarland standard using 7H9 complete media and serially diluted (10^−2^ and 10^−4^). A 0.1 ml aliquot of each culture mixture was inoculated onto 7H10 agar plates supplemented with 10% oleic acid, albumin, dextrose, and catalase (OADC). According to WHO recommendations, the critical concentrations of the tested drugs in 7H10 media were RIF, 1 mg/L; INH, 1.0 mg/L and 0.2 mg/L; ethambutol (EMB), 5 and 10 mg/L; streptomycin (STM), 2 mg/L and 10 mh/L; levofloxacin (LFX), 1 mg/L; and moxifloxacin (MXF), 0.5 mg/L. The critical concentrations of the tested drugs in 7H11 media were amikacin (AMK), 6 mg/L; kanamycin (KAN), 6 mg/L; capreomycin (CAP), 10 mg/L; ethionamide (ETO), 10 mg/L; and para-aminosalicylic acid (PAS), 8.0 mg/L. Resistance to pyrazinamide (PZA), 100 mg/L, was tested using the Bactec MGIT 960 system (Becton Dickinson Diagnostic Systems, Sparks, MD) as previously described (WHO, 2018). Growth on a control medium was compared to growth on the corresponding drug-containing medium to determine susceptibility. Cultures were incubated at 37°C for 3 weeks, and colony-forming units (CFUs) were counted. Resistance was defined as CFUs on the antibiotic quadrant over 1% of the CFUs on the antibiotic-free quadrant (Woods et al., 2011). The DST result was used to determine resistance or susceptibility. The tests were validated based on the susceptibility of *M. tuberculosis* H37Rv. Since STM was initially tested with RIF, INH, and EMB as first-line drugs, we categorized STM as a first-line drug. MDR is defined as an *M. tuberculosis* isolate that is resistant to at least INH and RIF. Pre-XDR is defined as an MDR isolate that is resistant to either fluoroquinolone (FQs; pre-XDR fluo) or at least one of the injectable drugs (pre-XDR inj; Banerjee et al., 2008). XDR was defined as MDR TB plus resistance to an FQ and at least one second-line injectable drug (SLID; WHO, 2007).

### 2.3 Whole-genome sequencing

*M. tuberculosis* isolates were subcultured on 7H11 medium, and genomic DNA was extracted using the cetyl-trimethyl-ammonium-bromide method (van Soolingen et al., 1991). The TruSeq DNA PCR-Free LT Sample Preparation Kit (Illumina, Inc., San Diego, CA, USA) was used to prepare paired-end libraries following the manufacturer's instructions. The average fragment size (500–600 bp) of the DNA libraries was checked by the Agilent 2100 Bioanalyzer in combination with the High Sensitivity DNA Kit. The concentration of the DNA libraries was measured by quantitative PCR with the KAPA Library Quantification Kit (Roche Sequencing Solutions, Inc., Pleasanton, CA, USA). Using the MiSeq Reagent Kit ver. 3 and an Illumina MiSeq system (Illumina, Inc., San Diego, CA, USA), the 24 pure DNA libraries were pooled (11 pM) and sequenced (600 cycles) with an expected coverage of 100X. We analyzed compensatory mutations in the *rpoA, rpoC*, and non-RRDR of the *rpoB* genes. Putative compensatory mutations were identified based on the following criteria: (1) the presence of non-synonymous mutations in the *rpoA, rpoB*, or *rpoC* genes in RIF-resistant isolates; (2) each putative compensatory mutation has been independently observed in at least two isolates (Liu et al., 2018).

### 2.4 Bioinformatics analysis

We conducted a dry laboratory analysis of WGS data (Behzadi and Ranjbar, 2019). Paired-end Illumina reads were checked using FastQC (www.bioinformatics.babraham.ac.uk/projects/fastqc/) for primary assessment of data quality, and any adapter fragment and low-quality reads were removed using Trimmomatic. Bowtie2 was used to map paired-end reads to the reference genome H37Rv (GenBank AL123456). The strict SNP filtering (closed SNP set) template within the BioNumerics 7.6.3 software was used to generate a list of high-quality, informative SNPs for each cluster. The SNP filtration was based on the following criteria: have total coverage of 5 reads, not contain ambiguous bases, not contain gaps, and not be within 12 base pairs of adjoining SNPs. Non-informative SNPs (identical in all samples) were also excluded. No genomic regions were specifically excluded from the analysis. The TB Profiler v.4.1.1 was used to assign lineages and predict gene mutations associated with drug resistance (Phelan et al., 2019). A phylogenetic tree was built from 3,453 identified SNP positions, excluding repetitive genomic regions (PE/PEE), using the maximum likelihood method with the Tamura-Nei model and 1,000 bootstrap replicates in MEGA 7.0. iTOL v6 (https://itol.embl.de) was used to annotate and visualize the phylogenetic tree. Isolates were defined as clustered or unique based on a genetic distance of ≤ 12 or > 12 SNPs, respectively. The sequence data were deposited in the National Center for Biotechnology Information Sequence Read Archive under BioProject ID PRJNA1141184. A minimum spanning tree was constructed using BioNumerics 7.6.3 software.

### 2.5 Statistical analysis

Descriptive statistics were performed on patients' demographics, including lineage, sex, age, previous treatment history, and clustering of MDR isolates. Logistic regression analysis was used to calculate statistical significance. Odd ratios with 95% confidence intervals (CI) were calculated, and variables with *P*-values < 0.05 were considered potential new risk factors. The odds ratio calculator from MedCalc Software Ltd. (version 20.013) was used for the statistical analysis.

### 2.6 Ethics statement

The Taiwan CDC Institutional Review Board approved this study (TwCDC IRB No. 106211). All procedures were conducted in accordance with applicable guidelines and regulations. As the study only involved archived isolates, written informed consent from participants was not required.

## 3 Results

### 3.1 Study population

**Figure 1:**
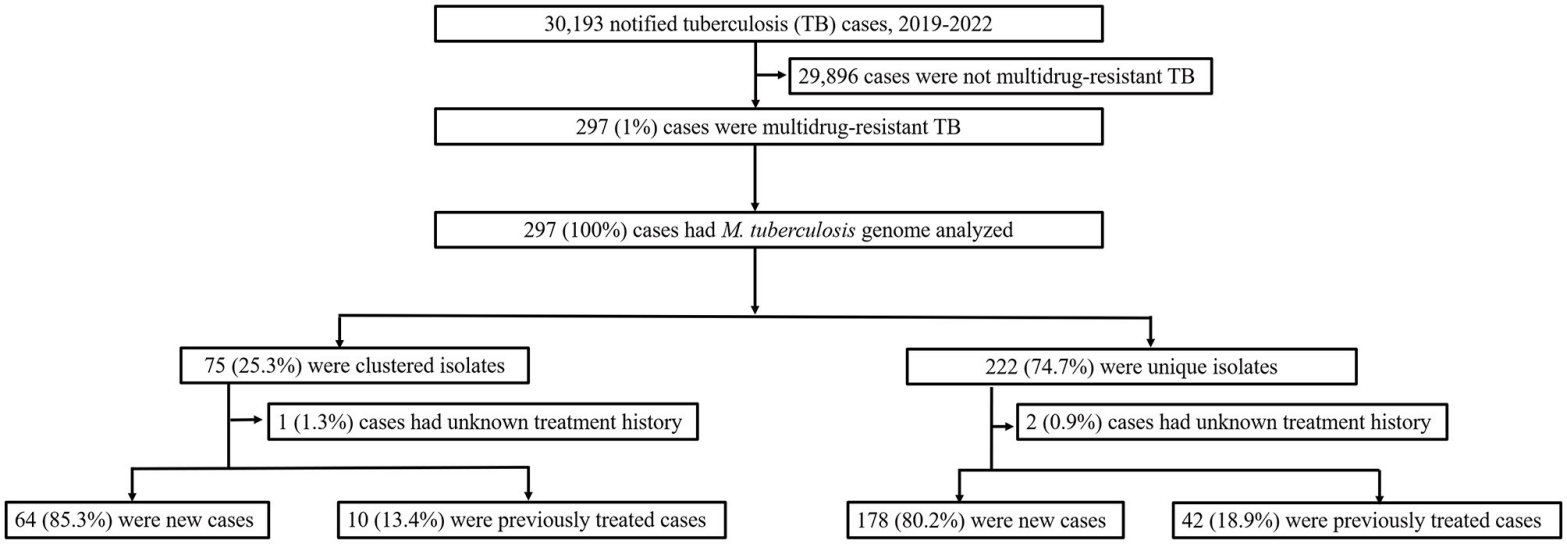
Classification of multidrug-resistant tuberculosis based on treatment history and genomic analysis. PTB, pulmonary tuberculosis; DST, drug susceptibility testing; MDR-TB, multidrug-resistant tuberculosis.

**Figure 2:**
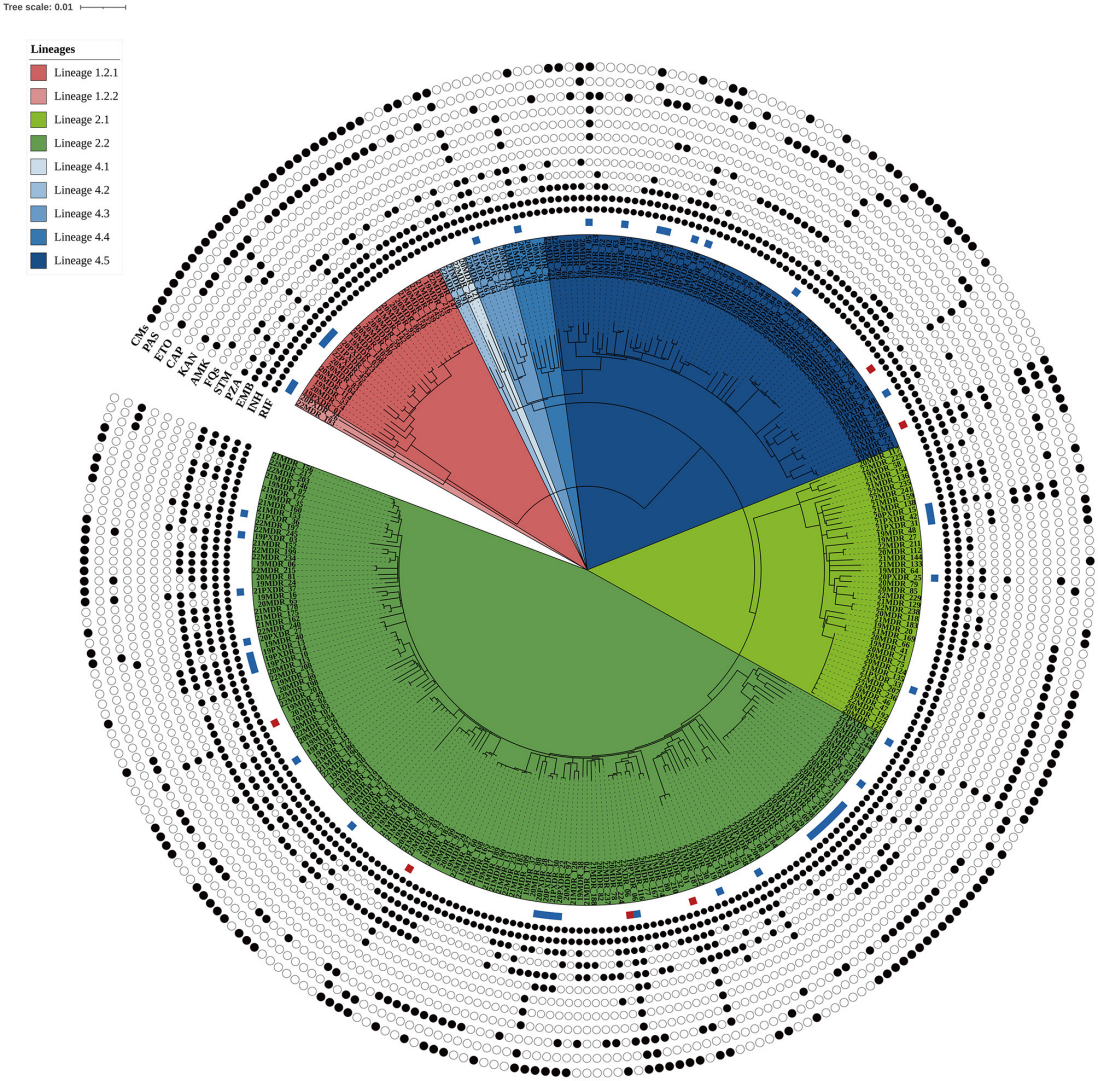
Maximum likelihood phylogenetic tree of the 297 MDR-TB isolates from Taiwan. The tree was constructed based on 3,453 genome-wide SNPs. Lineages are represented by different colored blocks. Mutations encoded resistance were represented by filled circles (presence of mutation) or empty circles (absence of mutations) icons. The square beside the nodes shows the presence of pre-extensively drug-resistant (pre-XDR, blue) and extensively drug-resistant (XDR, red) phenotypes among the multidrug-resistant tuberculosis strains. The figure was generated using iTOL v6 (https://itol.embl.de). RIF, rifampicin; INH, isoniazid; EMB, ethambutol; PZA, pyrazinamide; STM, streptomycin; FQs, fluoroquinolones; AMK, amikacin; KAM, kanamycin; CAP, capreomycin; ETO, ethionamide; PAS, para-aminosalicylic acid; CMs, compensatory mutations.

In this population-based study, 297 cases of MDR-TB accounted for 1.0% of the 30,193 TB cases from 2019 to 2022 (Figure 1). Of these 297 MDR-TB cases, 219 (73.7%) were male, and 78 (26.3%) cases were female, with a median age of 63 years (ranging from 50 to 76 years). Among the cases, 242 (81.5%) were new cases, 52 (17.5%) were previously treated cases, and 3 (1.0%) had an unknown treatment history. A maximum-likelihood phylogenetic tree was constructed based on 3,453 SNPs in non-repetitive regions of the studied isolates, which included 45 (15.2%) pre-XDR and 6 (2.0%) XDR-TB cases (Figure 2). Lineage 2 isolates were predominant, including 43 (14.5%) sublineage 2.1 (proto-Beijing) and 145 (48.8%) sublineage 2.2 (modern Beijing) isolates (Table 1). Data for all 297 MDR-TB cases are shown in Supplementary Table 3.

**Table 1:** Demographic and other factors associated with TB clustering ( ≤ 12 SNPs difference).

**Characteristic**	**Case (*n* = 297), No. (%)**	**Cluster (*n* = 75), No. (%)**	**Non-cluster (*n* = 222), No. (%)**	**OR (95% CI)**	***P*-value**
**Sex**					
Female	78 (26.3)	13 (17.3)	65 (29.3)	1.00	
Male	219 (73.7)	62 (82.7)	157 (70.7)	1.97 (1.01-3.84)	**0.04**
**Age**					
< 25	16 (5.4)	8 (10.7)	8 (3.6)	1.00	
25–44	44 (14.8)	9 (12.0)	35 (15.8)	0.25 (0.08–0.97)	0.03
45–54	41 (13.8)	12 (16.0)	29 (13.1)	0.41 (0.12–1.36)	0.15
55–64	56 (18.9)	19 (25.3)	37 (16.7)	0.51 (1.17–1.58)	0.25
≥65	140 (47.1)	27 (36.0)	113 (51.0)	0.24 (0.08–0.69)	0.01
**Case category**					
New	242 (81.5)	64 (85.3)	178 (80.2)	1.00	
Previously treated	52 (17.5)	10 (13.3)	42 (18.9)	0.66 (0.31–1.40)	0.23
Unknown	3 (1.0)	1 (1.3)	2 (0.9)	1.39 (0.12–15.60)	0.79
**Treatment outcome**					
Cured/Treatment completed	160 (53.9)	47 (62.7)	113 (50.9)	1.00	
Died	95 (32.0)	20 (26.7)	75 (33.8)	0.64 (0.35–1.17)	0.15
Unknown	42 (14.1)	8 (10.7)	34 (15.3)	0.57 (0.24–1.31)	0.18
**Genotype**					
Beijing	145 (48.8)	34 (45.3)	111 (50.0)	1.00	
Proto-Beijing	43 (14.5)	17 (22.7)	26 (11.7)	2.13 (1.04–4.39)	**0.04**
Non-Beijing	109 (36.7)	24 (32.0)	85 (38.3)	0.92 (0.51–1.67)	0.8
**Drug resistance mutations**					
w/o *rpoB* S450L	98 (33.0)	11 (14.7)	87 (39.2)	1.00	
w *rpoB* S450L	70 (23.6)	13 (17.3)	57 (25.7)	1.80 (0.76–4.30)	0.2
w *rpoB* S450L+CMs	129 (43.4)	51 (68.0)	78 (35.1)	5.17 (2.52–10.62)	**< 0.01**

OR, odds ratio; CI, confidence interval; CMs, compensatory mutations; w, with; w/o, without. Statistical significances are represented in bold.

### 3.2 MDR-TB clusters

**Figure 3:**
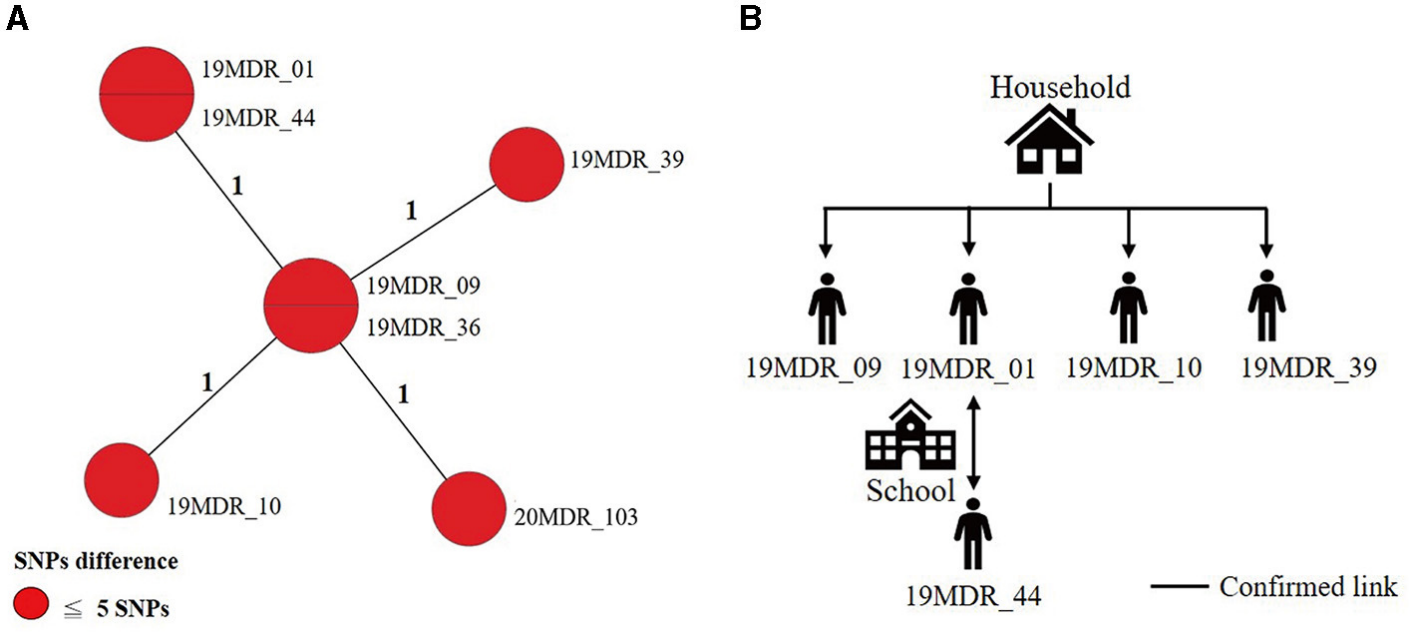
The minimum spanning tree of genomic cluster 10. **(A)** Minimum spanning tree of genomic cluster 10. **(B)** Putative transmission network based on epidemiological links. The numbers on the black lines are the difference in the number of SNPs between isolates. The first two digits of the case's identification indicated the year MDR-TB was notified.

**Figure 4:**
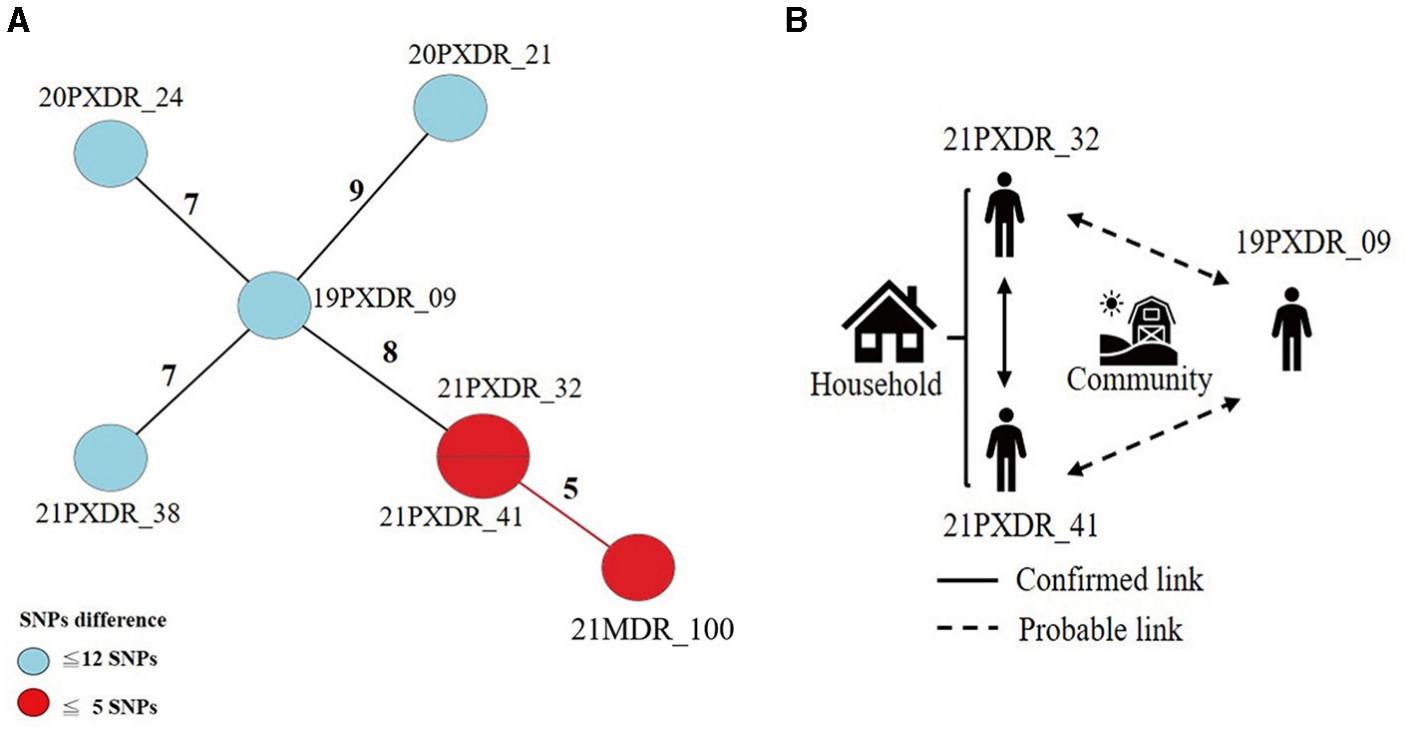
The results of the epidemiology investigation and genomic analysis of cluster 11. **(A)** Minimum spanning tree of genomic cluster 11 **(B)** Putative transmission network based on epidemiological links. The numbers on the black and red lines are the difference in the number of SNPs between isolates. The first two digits of the case's identification indicated the year MDR-TB was notified.

Using a 12-SNP cut-off, 25.3% (75/297) MDR-TB cases were grouped into 20 clusters, with cluster sizes ranging from 2 to 13 cases (Tables 1, 2). Cluster 04 was the largest cluster, with 84.6% (11/13) of cases originating from northern Taiwan, all of which had identical drug-resistance gene mutations, including *fabG1* t-8c and *rpoB* S450L, along with a compensatory mutation, *rpoC* E750D (Table 2 and Supplementary Table 2). The univariate analysis revealed a correlation between MDR-TB clusters and male sex. Individuals carrying the sublineage 2.1-proto-Beijing genotype had a higher risk of transmitting the infection (Table 1). Of the 20 clustered MDR-TB cases, 8 (40%) had definite epidemiological links. Minimum Spanning Tree (MST) analysis showed household and community links in clusters 10 and 11 (Figures 3, 4). In cluster 10, the SNP differences among the 7 cases ranged from 0 to 1 (Figure 3A). Cases 19MDR_01, 19MDR_09, 19MDR_10, and 19MDR_39 lived in the same household, while cases 19MDR_01 and 19MDR_44 attend the same school (Figure 3B). However, 19MDR_36 and 20MDR_103 had no known epidemiological links with other cases in the cluster. In cluster 11, cases 21PXDR_32, 21PXDR_47, and 19PXDR_09 were pre-XDR TB cases with SNP differences ranging from 0 to 8 (Figure 4A) and were from a high-burden village with presumptive infection from the community (Figure 4B). Cases 21PXDR_32 and 21PXDR_41 were household members (Figure 4B). Although the SNP difference between cases 20MDR_100 and 21PXDR_32 was 5, no epidemiological links were recalled.

**Table 2:** Characteristics of MDR-TB clusters based on whole genome sequencing analysis.

**Clusters**	**Number of cases in a cluster**	**Lineage**	**Median age (years [IQR])**	**Number of patients living in the same region**	**Number of new cases**	**Treatment outcome (number of patients)**	**Nature of epidemiological link**
Cluster 04	13	2.1	67 (54–76)	11	13	Cure or completed (10), died (2), unknown (1)	Unknown
Cluster 19	8	2.2	65 (50–77)	5	8	Cure or completed (5), died (2), unknown (1)	Unknown
Cluster 10	7	4.5	52 (25–64)	6	7	Cure or completed (6), died (1)	Household (5), social (school [2])
Cluster 11	7	2.2	40 (34–46)	2, 5^*^	5	Cure or completed (5), died (2)	Household (2), social (resident community [3])
Cluster 01	5	1.2.1	58 (56–58)	5	4	Cure or completed (3), died (1), unknown (1)	Unknown
Cluster 05	4	4.5	55 (48–59)	4	2	Cure or completed (2), died (2)	Unknown
Cluster 13	4	2.2	61 (56–66)	3	3	Cure or completed (3), died (1)	Unknown
Cluster 20	3	2.2	56 (53–64)	3	3	Cure or completed (3)	Unknown
Other clusters^**^	24	2.1 or 2.2 or 4.5	59 (52–75)	ND	19	Cure or completed (11), died (9), unknown (4)	Unknown

^*^Five cases were from the eastern region, and two cases were from the central region.

^**^Genomic cluster includes two independent MDR-TB strains.

ND, not determined.

### 3.3 Compensatory mutations

We identified putative compensatory mutations in the *rpoA, rpoC*, or non-RRDR of the *rpoB* genes in 161 (54.2%) MDR isolates. The most predominant mutation was *rpoC* V483G (17/161, 10.6%), one of the high-probability compensatory mutations (HCMs) identified by Comas et al. (2011), followed by the *rpoC* A172V (14/161, 8.7%) and *rpoC* E750D (14/161, 8.7%) mutations (Supplementary Table 1). The putative compensatory mutation *rpoC* E750D was not previously reported and was found only among sublineage 2.1 isolates (Supplementary Table 1). We observed that 43.43% (129/297) of *M. tuberculosis* isolates harbored concurrent compensatory mutations with *rpoB* S450L mutation. Notably, these mutations were significantly associated with clustering (Table 1). In addition, the *rpoC* E750D in sublineage 2.1 and the *rpoC* D485Y/*rpoC* E1140D in sublineage 2.2 were associated with MDR-TB transmission (Table 3).

**Table 3:** Univariable analysis of MDR-TB strains harbored *rpoB* S450L with compensatory mutations associated with clustering.

**Gene catalog**	**Case (*n* = 129), No. (%)**	**Cluster (*n* = 51), No. (%)**	**Non-cluster (*n* = 78), No. (%)**	**OR (95% CI)**	***P*-value**
*rpoC* V483G	15 (11.6)	4 (7.8)	11 (14.1)	0.52 (0.16–1.72)	0.29
*rpoC* E750D	13 (10.1)	13 (25.5)	0 (0.0)	55.1 (3.19–950.72)	**< 0.01**
*rpoC* N698S	9 (7.0)	4 (7.8)	5 (6.4)	1.24 (0.92–4.87)	0.76
*rpoC* A172V	8 (6.2)	0 (0.0)	8 (10.3)	0.08 (0.004–1.43)	0.09
*rpoC* D485Y + *rpoC* E1140D	8 (6.2)	7 (13.7)	1 (1.3)	12.3 (1.46–102.86)	**0.02**
*rpoC* H525Q	5 (3.9)	0 (0.0)	5 (6.4)	0.13 (0.01–2.40)	0.17
*rpoC* V483A + *rpoC* A172V	8 (6.2)	5 (9.8)	3 (3.8)	2.71 (0.62–11.9)	1.18
*rpoC* E750G	4 (3.1)	0 (0.0)	4 (5.1)	0.16 (0.01–3.05)	0.22
*rpoC* L449V	4 (3.1)	4 (7.8)	0 (0.0)	14.87 (0.78–282.46)	0.07
*rpoC* P1040S	4 (3.1)	1 (2.0)	3 (3.8)	0.5 (0.05–4.94)	0.55
*rpoB* F503S	3 (2.3)	2 (3.9)	1 (1.3)	3.14 (0.28–35.60)	0.36
*rpoB* G890C	3 (2.3)	3 (5.9)	0 (0.0)	11.33 (0.57–224.13)	0.11
*rpoB* I491V	3 (2.3)	2 (3.9)	1 (1.3)	3.14 (0.28–35.60)	0.36
*rpoC* L516P	3 (2.3)	0 (0.0)	3 (3.8)	0.21 (0.01–4.14)	0.30
Others^*^	39 (30.3)	6 (11.9)	33 (42.4)	0.18 (0.07–0.48)	**< 0.01**

^*^Gene catalog of compensatory mutations occurred in two independent MDR-TB strains.

OR, odds ratio.

Statistical significances are represented in bold.

## 4 Discussion

To contain and mitigate ~80% of new MDR-TB cases confirmed annually in Taiwan, we applied WGS analysis alongside epidemiological investigation to delineate the putative transmission network and guide public health responses. We found that the MDR-TB clustering rate was 25.3%, consisting of 20 clusters with two outbreaks. In comparison, the clustering rates were 20 and 15% in the US and UK, respectively (Moonan et al., 2013; Anderson et al., 2014). The Beijing genotype was predominant, with 39.5% of the proto-Beijing genotype isolates found in clusters (Table 1). Our results recapitulated that MDR isolates harboring the low fitness cost *rpoB* S450L mutation and compensatory mutations in the *rpoC* gene have an impact on MDR-TB transmission (de Vos et al., 2013). Particularly, sublineage 2.1 MDR isolates harboring the *rpoB* S450L and a compensatory mutation in *rpoC* E750D had a heightened risk of clustering.

Our results supported previous findings indicating that lineage 2 isolates were the most common in MDR-TB clusters (Yang et al., 2017; Yin et al., 2022; Xiao et al., 2023). Previous studies revealed that lineage 2 isolates displayed greater virulence than other lineages in East Asia. Specifically, lineage 2 isolates induce reduced lower levels of pro-inflammatory cytokines TNF and IL-12p40 and proliferate rapidly within monocyte-derived macrophages (Sarkar et al., 2012; Smith et al., 2022). These characteristics facilitate immune evasion, contributing to the widespread transmission of lineage 2 isolates in densely populated East Asian regions (Borrell and Gagneux, 2009; Chai et al., 2020; Allué-Guardia et al., 2021; Chandra et al., 2022). Notably, we identified 14.5% of isolates as belonging to sublineage 2.1, a prevalence comparable to that in southern China, Guangxi (9.3%), and Hainan Island (27.2%; Liang et al., 2023; Wang et al., 2023), but not in central China, Ningbo (0.9%), or Japan (2.35%; Che et al., 2024; Yokoyama et al., 2010). This suggests that sublineage 2.1 exhibits considerable geographic variability, potentially influenced by local environmental conditions and specific genetic adaptations that may confine its epidemic presence to certain areas. Additionally, research has shown that sublineage 2.1 isolates in MDR or XDR-TB cases have been linked to transmission in Thailand, although the reasons remain unclear (Srilohasin et al., 2020). Investigating host immune responses to sublineage 2.1 strains might further clarify the relationship between geographic distribution and epidemiological patterns.

Our study demonstrated a significant association between *rpoC* compensatory mutations and MDR-TB clustering. Previous studies have shown that RIF-resistant *M. tuberculosis* isolates with concurrent compensatory mutations significantly impact increased transmission rates (de Vos et al., 2013; Casali et al., 2014; Li et al., 2016; Merker et al., 2018; Gygli et al., 2021; Goig et al., 2023). A study in South Africa showed that clustered *M. tuberculosis* isolates had a significantly higher prevalence of *rpoC* mutants than non-clustered isolates (30.8 vs. 9.4%). Although the association between *rpoC* gene mutations and MDR-TB dissemination has been hypothesized, the transmission mechanism remains unclear (de Vos et al., 2013). We observed a high-probability compensatory mutation, *rpoC* V483G, in 10.6% of MDR isolates, which was prevalent in high-burden MDR isolates (Comas et al., 2011). The compensatory mutation in *rpoC* V483G could restore the significant fitness cost caused by the low fitness *rpoB* S450L under stringent growth conditions (Song et al., 2014).

When exposed to RIF, MDR isolates carrying the *rpoB* S450L and F503S mutations demonstrated increased *in vitro* growth (Zhong et al., 2010). Furthermore, studies have indicated that the presence of the *rpoB* S450L mutation, along with additional compensatory mutations, is associated with higher transmission rates (Conkle-Gutierrez et al., 2024). A study from Russia further demonstrated that MDR isolates with the *rpoB* S450L mutation and the compensatory mutation, *rpoB* E761D, could enhance transmission (Casali et al., 2014).

In addition, Brunner et al. showed that isolates harboring the *rpoB* S450L mutation along with compensatory mutations promoted lineage and cluster formation during *in vitro* growth (Brunner et al., 2024). These compensatory mutations likely facilitate the clustering of MDR isolates with *rpoB* S450L, possibly due to their effects on protein structure. The *rpoB* mutation might disrupt the structural confirmation and interactions among the β′, β, and α subunits of RNA polymerase, negatively impacting growth and RNA transcription. However, compensatory mutations could reverse these impacts (Gagneux et al., 2006; Brandis et al., 2012; Li et al., 2016; Brunner et al., 2024).

Previous studies suggest that the survival and evolution of MDR isolates may involve epistatic interactions between various drug-resistant and compensatory mutations (Phillips, 2008; Borrell and Gagneux, 2011). The epistatic interactions between specific mutations, such as *rpoB* S450L and *rpoC* compensatory mutations, particularly novel and high-confidence mutations, warrant further investigation.

Compensatory mutations could be lineage-specific. The lineage markers, *tlyA* N236K and *rpoC* G594E, were identified in lineage 4.6.2 (Cameroon genotype) and lineage 4.1.2 (Haarlem lineage), respectively (Comas et al., 2011; Walker et al., 2018). In our study, all lineage 1.2.1 and 1.2.2 isolates carried the compensatory mutation *rpoC* A172V, consistent with findings in Indo-Oceanic genotype EAI isolates in India (Comas et al., 2011; Advani et al., 2019). A cluster-specific gene marker has been used in *Shigella* and enteroinvasive *Escherichia coli* (EIEC) for epidemiological and diagnostic inquiries (Zhang et al., 2021). Our research showed that compensatory mutations in the *rpoC* gene could prompt clustering and transmission (Table 3 and Supplementary Table 2), indicating their potential use as epidemiological gene markers or lineage-specific markers to predict putative clusters. Compensatory mutations may serve as prognostic indicators, facilitating epidemic alerts and responses to MDR-TB clustering and outbreaks.

The strength of this study lies in its population-based cohort design, which effectively demonstrates MDR-TB clustering. However, there are some limitations to be considered. First, the lack of detailed epidemiological data hinders the ability to definitively identify true outbreaks among cases within the same cluster. Additionally, the lack of spatial transmission analysis limits our comprehensive understanding of the transmission dynamics. To address this, we plan to employ Geographic Information Systems (GIS) to conduct spatial analysis to enhance molecular epidemiological investigations.

## 5 Conclusion

In conclusion, this study provides the first report on the population-based genomic epidemiological analysis of MDR-TB in Taiwan, revealing that 25.3% of cases were clustered. The sublineage 2.1-proto-Beijing genotype MDR isolates were identified as having a high risk of transmission. We found that 54.2% of isolates harbored compensatory mutations in the *rpoC* gene and non-RRDR regions of the *rpoB* gene.

Notably, proto-Beijing genotype isolates with concurrent *rpoB* S450L/*rpoC* E750D mutations and modern Beijing genotype isolates harboring *rpoC* D485Y/*rpoC* E1140D mutations were significantly associated with MDR clusters.

We propose that specific compensatory mutations could serve as epidemiological markers to detect clusters and putative outbreaks. Overall, we modernized and strengthened laboratory services and surveillance by systemically integrating WGS-based analysis with public health investigations to elucidate the genetic basis of MDR-TB clusters.

## Data Availability

The datasets presented in this study can be found in online repositories. The names of the repository/repositories and accession number(s) can be found in the article/Supplementary material.
