# Supplementary material for: Multidrug-resistant tuberculosis clusters and transmission in Taiwan: a population-based cohort study
Source: Front Microbiol. 2024 Sep 18;15:1439532. doi: 10.3389/fmicb.2024.1439532 (PMC11445003; doi:10.3389/fmicb.2024.1439532)
Supplement: Supplementary file 1 [file Table_1.DOCX]

Table S1. Catalog of compensatory mutations of the *rpo* genes

| Compensatory mutations | sublineages (No.) | HCM | No. of isolates | % (n=161) |
| --- | --- | --- | --- | --- |
| *rpoC* V483G | 2.1(5), 2.2(11), 4.5(1) | ○ | 17 | 10.6 |
| *rpoC* A172V | 1.2.1(12), 1.2.2 (2) | X | 14 | 8.7 |
| *rpoC* E750D | 2.1 (14) | X | 14 | 8.7 |
| *rpoC* N698S | 2.1(1), 2.2(2), 4.5(6) | ○ | 9 | 5.6 |
| *rpoC* A172V+ *rpoC* V483A | 1.2.1(8) | X | 8 | 5.0 |
| *rpoC* D485Y + *rpoC* E1140D | 2.2 (8) | X | 8 | 5.0 |
| *rpoB* R167C | 2.2(1), 4.5(4) | X | 5 | 3.2 |
| *rpoC* H525Q | 2.2(4), 4.5(1) | X | 5 | 3.2 |
| *rpoC* E750G | 2.2(1), 2.3(1), 4.5(2) | X | 4 | 2.5 |
| *rpoC* L449V | 2.1(2), 4.5(2) | ○ | 4 | 2.5 |
| *rpoC* P1040S | 4.5 (4) | X | 4 | 2.5 |
| *rpoB* F503S | 2.2 (3) | X | 3 | 1.9 |
| *rpoB* G890C | 2.2 (3) | X | 3 | 1.9 |
| *rpoB* I491V | 2.2 (3) | X | 3 | 1.9 |
| *rpoB* K832E | 2.2(2), 4.5(1) | X | 3 | 1.9 |
| *rpoB* Q172R | 2.2 (3) | X | 3 | 1.9 |
| *rpoC* E1140D | 2.2 (3) | X | 3 | 1.9 |
| *rpoC* L516P | 2.2(2), 4.5(1) | X | 3 | 1.9 |
| *rpoC* N416S | 2.2 (3) | X | 3 | 1.9 |
| *rpoC* S561P | 2.2 (3) | X | 3 | 1.9 |
| *rpoC* V483A | 2.2(2), 4.5(1) | ○ | 3 | 1.9 |
| *rpoC* W484G | 2.1(1), 2.2(2) | ○ | 3 | 1.9 |
| r*poC* A1037V | 2.2 (2) | X | 2 | 1.2 |
| *rpoC* V1039A | 2.2 (2) | X | 2 | 1.2 |
| *rpoB* E481A | 2.2(1), 4.5(1) | X | 2 | 1.2 |
| *rpoB* I491T | 2.2 (2) | X | 2 | 1.2 |
| *rpoB* P45L + rpoC A172V | 1.2.1(8) | X | 2 | 1.2 |
| *rpoB* V534A | 2.2 (2) | X | 2 | 1.2 |
| *rpoB* V534M | 2.2 (2) | X | 2 | 1.2 |
| *rpoB* Y564H | 2.1(1), 4.3(1) | X | 2 | 1.2 |
| *rpoC* A172V + *rpoC* T589A | 1.2.1(2) | X | 2 | 1.2 |
| *rpoC* A492P | 2.2(1), 4.5(1) | X | 2 | 1.2 |
| *rpoC* E518D | 2.2(1), 4.5(1) | X | 2 | 1.2 |
| *rpoC* F452L | 2.2 (2) | X | 2 | 1.2 |
| *rpoC* G433S | 2.2(1), 4.5(1) | X | 2 | 1.2 |
| *rpoC* G594E | 4.1 (2) | X | 2 | 1.2 |
| *rpoC* M312V | 2.2 (2) | X | 2 | 1.2 |
| *rpoB* F503S + *rpoC* A172V | 1.2.1(1) | X | 1 | 0.6 |
| *rpoB* P45L | 2.2 (1) | X | 1 | 0.6 |
| *rpoC* A172V + *rpoB* V534M | 1.2.1(1) | X | 1 | 0.6 |
| *rpoC* A172V + *rpoC* D485Y | 1.2.1(1) | X | 1 | 0.6 |
| *rpoC* L516P + *rpoC* E1140D | 2.2 (1) | X | 1 | 0.6 |
| *rpoC* V483G + *rpoC* E1140D | 2.2 (1) | X | 1 | 0.6 |

Abbreviations: HCM, high-probability compensatory mutations
